# Measuring Mindfulness: Assessing the Utility of the FFMQ in the Older Veterans With Dementia and Their Caregivers

**DOI:** 10.1093/geroni/igab046.3712

**Published:** 2021-12-17

**Authors:** Brian Clawson, Phoebe Block, Daniel Durkin, Amber Collins, Lindsey Jacobs, Michelle Hilgeman

**Affiliations:** 1 Tuscaloosa VA, Tuscaloosa, Alabama, United States; 2 Battle Creek VA Medical Center, Battle Creek, Michigan, United States; 3 The University of Mississippi, University, Mississippi, United States; 4 Tuscaloosa VA Medical Center, Tuscaloosa, Alabama, United States

## Abstract

Mindfulness is increasingly popular as a low cost, convenient, and accessible way to address mental health and chronic health conditions. Despite its popularity, best practices in measuring mindfulness in clinical settings and intervention research are still being defined. The Five Facet Mindfulness Questionnaire (FFMQ-15) measures mindfulness traits; however, its use has been limited in older adults, those with dementia, and caregivers. Method: Caregivers (N=82) and veterans with dementia (N=62) enrolled in a randomized pilot intervention study completed the 15-item FFMQ at baseline, 6-month, and 12-month assessments. Veterans were mostly male (98%), White/Caucasian (65%), and living with a partner/spouse (79%). Caregivers (M=65 years old) were mostly female (89%) and White/Caucasian (66%). FFMQ response options were simplified to a 3-point Likert-scale for individuals with dementia (0=rarely true to 2=often true). Results: Internal consistency statistics (Cronbach’s alphas) at the scale-level were acceptable among caregivers at baseline and 6-months (.71-.75) but questionably reliable at 12-months (.59, N=46). For individuals with dementia, the simplified version of the FFMQ (with 3 response options) achieved questionable reliability at baseline (.57, N=56) and 6-months (.67, N=32), but improved to acceptable at 12-month assessments (.75, N=15), after significant attrition. Conclusion: Researchers should apply caution when using the FFMQ total score with caregivers and those with cognitive impairments. Though simplified response options eased administration, utility of the tool may be limited in those who are more impaired. Before mindfulness measures can be used meaningfully, reliability of available tools like the FFMQ-15 need to be examined in more diverse samples.

